# Accurate confidence intervals for risk difference in meta-analysis with rare events

**DOI:** 10.1186/s12874-020-00954-8

**Published:** 2020-04-30

**Authors:** Tao Jiang, Baixin Cao, Guogen Shan

**Affiliations:** 1 School of Statistics and Mathematics, and School of Business, Zhejiang Gongshang University, Hangzhou, Zhejiang, China; 2 School of Mathematical Sciences, Nankai University, Tianjin, China; 3 Epidemiology and Biostatistics Program, School of Public Health, University of Nevada Las Vegas, Las Vegas, USA

**Keywords:** Binary outcome, Confidence interval, Importance sampling, Meta-analysis, Rare events

## Abstract

**Background:**

Meta-analysis provides a useful statistical tool to effectively estimate treatment effect from multiple studies. When the outcome is binary and it is rare (e.g., safety data in clinical trials), the traditionally used methods may have unsatisfactory performance.

**Methods:**

We propose using importance sampling to compute confidence intervals for risk difference in meta-analysis with rare events. The proposed intervals are not exact, but they often have the coverage probabilities close to the nominal level. We compare the proposed accurate intervals with the existing intervals from the fixed- or random-effects models and the interval by Tian et al. (2009).

**Results:**

We conduct extensive simulation studies to compare them with regards to coverage probability and average length, when data are simulated under the homogeneity or heterogeneity assumption of study effects.

**Conclusions:**

The proposed accurate interval based on the random-effects model for sample space ordering generally has satisfactory performance under the heterogeneity assumption, while the traditionally used interval based on the fixed-effects model works well when the studies are homogeneous.

## Background

Meta-analysis is a useful statistical tool in medical research to evaluate treatment effect by analyzing outcomes from multiple clinical trials. The estimated treatment effect from meta-analysis is always more reliable and accurate than the estimate from one selected study among the available studies. In early phase clinical trials to study safety of a new drug, rare events are very common [1]. In meta-analysis for such data, Vandermeer et al. [1] pointed out that the traditionally used asymptotic point estimates and confidence intervals could be substantially different from the results using exact methods under the exact conditional framework [2]. It is well known that asymptotic approaches often do not have satisfactory performance when outcome is extreme or sample size is small.

Multiple methods have been developed for meta-analysis with rare events over decades [3, 4]. The fixed-effects models are conveniently used in practice, such as the Mantel-Haenszel method [5]. When one or both groups in a study have zero events, a continuity correction is often needed in order to estimate risk ratio or odds ratio, but the traditional correction by adding 0.5 may lead to undesirable influence on the analysis results as pointed out by Sweeting *et al* [6]. Later, they developed a continuity correction method by adding a float value based on the size of each group to improve the coverage probability. Multiple follow-up articles discussed this issue whether or not a small value should be added to studies with rare events in data analysis [7,8]. Kuss et al. [8] suggested using a beta-binomial model to avoid adding arbitrary values to each cell in data analysis. Recently, Tian et al. [9] proposed a simple and effective method for confidence interval calculation without artificial continuity correction. The confidence intervals from each study were weighted to construct an overall interval from simulation studies under the fixed-effects model. Their developed confidence intervals were shown to have better coverages when the events are rare, but the length of their intervals could be much longer than others.

In contrast to fixed-effects models, the treatment effect in the random-effects model is assumed to follow a normal distribution. DerSimonian and Laird [10] proposed a random-effects model by including a random study effect to account for the variation of study population or study design. The statistical software R package *meta* can be used to compute confidence intervals for the fixed-effects model and random-effects model [11]. Recently, Bakbergenuly and Kulinskaya [12] suggested the generalized linear mixed models (GLMMs) in meta-analysis to include the correlation between point estimate and its variance estimate in data analysis.

The aforementioned exact conditional approach assumes both marginal totals in each study fixed [2]. It is reasonable to assume that the numbers of participant in each treatment group are fixed. It is not usual that a repeated study has the same total number of events as the observed study. The exact one-sided limit by Buehler [13] follows the study design with sample size in each treatment group fixed [14*–*16]. However, it is too computationally intensive to generate all possible samples in meta-analysis with binary outcome [17].

In this article, we propose using importance sampling to construct confidence interval for risk difference in meta-analysis with rare events. We apply the importance sampling method described by Lloyd and Li [18] to compute the profile confidence limit proposed by Kabaila and Lloyd [19]. Importance sampling methods have been studied by many researchers with regards to coverages of confidence intervals [20*,*^21^]. Importance sampling does not require to enumerate all possible samples [19]. This approach simulates samples from the distribution estimated from the observed data. Importance sampling has to be used in conjunction with a designated statistic to order the limits of simulated samples. We consider the existing intervals from the fixed-effects and random-effects models as designated statistics in this article.

The rest of this article is organized as follows. In “Methods” section, we describe the fixed-effects and random-effects models to estimate confidence intervals for risk difference. We then introduce importance sampling for interval calculation. In “Results” section, we use an example from 18 schizophrenia clinical trials to illustrate the application of the proposed intervals, and then compare the proposed intervals with the existing intervals with regards to coverage probability and average length. In “Conclusions” section, we provide some remarks on data analysis for meta-analysis with rare events.

## Methods

For meta-analysis with binary outcome, data can be organized in a *K*×4 table, where *K* is the number of studies (Table 1). Each row represents the results from a parallel study with the number of events and the number of non-events in the new treatment group and the control group, respectively. Let the two treatment groups be indexed by 0 and 1 for the control and the new treatment, respectively. Suppose *X*_*ijr*_ is the number of participants having *r* events from the treatment *j* in *i*th study, where *i*=1,2,⋯,*K*,*j*=0,1, and *r*=0,1. For studies with rare events, *X*_*ij*1_ is often very small. Let *n*_*ij*_=*X*_*ij*1_+*X*_*ij*0_ be the total number of participants from the treatment *j* in the *i*th study, and *N*_1_=(*n*_11_,*n*_21_,⋯,*n*_*K*1_) and *N*_0_=(*n*_10_,*n*_20_,⋯,*n*_*K*0_) be the sample sizes for the new treatment group and the control group, respectively. Suppose *p*_*j*_ is the event rate of the treatment *j*. Given the sample size *n*_*ij*_, the number of responses among these participants, *X*_*ij*1_, follows a binomial distribution, *B*(*n*_*ij*_,*p*_*j*_). We assume that each study is independent from each other, and the two groups within each study are independent from each other as well. The parameter of interest here is the risk difference between the treatment group and the control group,

$$\Delta=p_{1}-p_{0}.$$

**Table 1:** Data from *K* independent studies with binary outcome

Study	Treatment group		Control group	
	Events	Non-events	Events	Non-events
1	*X*_111_	*X*_110_	*X*_121_	*X*_120_
2	*X*_211_	*X*_210_	*X*_221_	*X*_220_
⋯	⋯	⋯	⋯	⋯
K	*X*_*K*11_	*X*_*K*10_	*X*_*K*21_	*X*_*K*20_

We first review the existing methods to construct two-sided confidence intervals for *Δ* in “Intervals based on fixed or random-effects model” section, and then develop accurate intervals in “Accurate intervals” section.

### Intervals based on fixed or random-effects model

We first consider the fixed-effects model to calculate confidence interval for *Δ*. Under the study homogeneity assumption, the treatment effect in each study is assumed to be the same,

$$\Delta_{i}=\mu,$$

where *μ* is the treatment effect. In the *i*th study, the risk difference *Δ*_*i*_ is estimated as

$$\widehat \Delta_{i}=\hat p_{i1}-\hat p_{i0},$$

where $\hat p_{ij}=X_{ij1}/n_{ij}$ is the estimated rate of the treatment *j* in the *i*th study. The variance is estimated as $s_{i}^{2}=\sum _{j=0}^{1} \frac {\hat p_{ij}(1-\hat p_{ij})}{n_{ij}}$ from two independent proportions. The weight for the *i*th study is

$$w_{i}=\frac{n_{i1}n_{i0}}{n_{i1}+n_{i0}}\frac{1}{\sum_{i=1}^{K} \frac{n_{i1}n_{i0}}{n_{i1}+n_{i0}}},$$

where $\sum _{i=1}^{K} \frac {n_{i1}n_{i0}}{n_{i1}+n_{i0}}$ is the factor to standardize the weight values, with $\sum _{i=1}^{K} w_{i}=1$. It is easy to show that *w*_*i*_ is an increasing function of *n*_*i*1_ (*n*_*i*0_) when *n*_*i*0_ (*n*_*i*1_) is fixed.

The overall weighted treatment effect using the fixed-effects model is calculated as

$$\widehat\Delta_{F}={\sum_{i=1}^{K} w_{i} \widehat\Delta_{i} }.$$

and its variance is estimated as

$$\widehat {SE}_{F}^{2}={\sum_{i=1}^{K} w_{i}^{2} s_{i}^{2}}.$$

The standardized statistic $\widehat \Delta / \widehat {SE}_{F}$ follows the standard normal distribution asymptotically when *Δ*=0. Therefore, the asymptotic confidence interval for *Δ* based on the fixed-effects model (the F interval) at the nominal level of 100(1−*α*)*%* is

1 $$ CI_{F}=(\widehat\Delta_{F}-z_{1-\alpha/2} \widehat {SE}_{F},\widehat\Delta_{F}+z_{1-\alpha/2} \widehat {SE}_{F}),   $$

where *z*_*a*_ is the *a*th quantile of the standard normal distribution.

In the observation of study heterogeneity which could be caused by study population or study design or influential covariates, DerSimonian and Laird [10] proposed using the random-effects model to include the study random effect in the model as

$$\Delta_{i}=\mu + u_{i},$$

where *u*_*i*_ is the deviation of the *i*th study from the population mean *μ*, and it follows a normal distribution. Let *v*_*i*_ be the weight of the *i*th study from the fitted random-effects model. Then, the weighted treatment effect and its variance are $\widehat \Delta _{R}={\sum _{i=1}^{K} v_{i} \widehat \Delta _{i} }$, and $\widehat {SE}_{R}^{2}={\sum _{i=1}^{K} v_{i}^{2} s_{i}^{2}} $, respectively. It follows that the asymptotic confidence interval for *Δ* using the random-effects model (the R interval) is computed as

2 $$ CI_{R}=(\widehat\Delta_{R}-z_{1-\alpha/2} \widehat {SE}_{R},\widehat\Delta_{R}+z_{1-\alpha/2} \widehat {SE}_{R}),   $$

It can be seen that the difference between *CI*_*F*_ and *CI*_*R*_ is the weights used in the treatment effect and its variance calculation. The F interval and the R interval can be computed by using the function *metabin* from the statistical software package *meta* [11*,*^22^]. In the *metabin* function, we use *MH*.*exact*=*TRUE* in the option with no continuity correction in the estimates.

### Accurate intervals

Exact confidence limit by Buehler [13] for *Δ* is preferable, but it is computationally intensive to save all the possible samples in meta-analysis with sample size *n*_*ij*_ fixed. For this reason, we consider importance sampling (IS) to construct accurate intervals for *Δ* by simulating samples from the distribution estimated from the observed data to make statistical inference. Importance sampling has been applied to many important medical research areas that often only have one nuisance parameter (e.g., the proportion difference in a parallel study [21*,*^23^]). We extend the application of IS to meta-analysis with multiple nuisance parameters in confidence interval calculation. The intervals computed using importance sampling are accurate with coverage close to the nominal level. In addition, importance sampling has the computational advantage over exact methods [19].

The calculation of the IS intervals has to be used in conjunction with a designated statistic for the interval ordering. Let *T* be the considered designated statistic. Suppose **p**_0_=(*p*_10_,*p*_20_,⋯,*p*_*K*0_) is the probability vector of the control group, where *p*_*i*0_ is the probability of the control group in the *i*th study. The accurate upper limit based on the designated statistic *T* is computed as the supremum of *Δ* such that

3 $$ G(\Delta)=P\Big(T(\mathbf{Y})\leq T(\mathbf{y}_{\text{\textbf{obs}}})\ |\ \Delta,\hat{\mathbf{p}}_{0}(\Delta)\Big)>\frac{\alpha}{2},   $$

where **y**_**obs**_ is the observed data, **Y** is data from the simulated data set, and $\hat {\mathbf {p}}_{0}(\Delta)$ is the maximum likelihood estimate of **p**_0_ given *Δ*.

Suppose we simulate *B* data sets from independent binomial distributions with the probabilities using $\widehat {\Delta }^{*}$ and $\widehat {\mathbf {p}}_{0}(\Delta ^{*})$ estimated from the observed data **y**_**obs**_. For studies with double zeros, although their estimated risk differences are zero, sample sizes from such studies are still valuable information in estimating the overall *Δ* and it confidence intervals [24]. Sample sizes from all studies including the ones with double zeros are used in the proposed method. The number of events are simulated from binomial distributions with the probabilities of $\widehat {\mathbf {p}}_{0}(\Delta ^{*})$.

The designated statistic of each simulated data set is computed, and compared with *T*(**y**_**obs**_). The set of *T*(**Y**)≤*T*(**y**_**obs**_) equals to *Ω*_*T*_(**y**_**obs**_)={**Y**:*T*(**Y**)≤*T*(**y**_**obs**_)}. Let the size of *Ω*_*T*_(**y**_**obs**_) be *B*_1_ with data: $\phantom {\dot {i}\!}\mathbf {Y}_{1}, \cdots, \mathbf {Y}_{B_{1}}$. Then, the upper limit in Eq. 3 can be rewritten as the supremum of *Δ* such that

$$\widehat G(\Delta)=\frac{1}{B}\sum_{b=1}^{B_{1}} \frac{f(\mathbf{Y}_{\mathbf{b}}|\Delta,\widehat{\mathbf{p}}_{0}(\Delta))}{f(\mathbf{Y}_{\mathbf{b}}|\widehat{\Delta}^{*},\widehat{\mathbf{p}}_{0}(\Delta^{*}))}>\frac{\alpha}{2},$$

where *f*(**Y**_**b**_) is the probability density function of **Y**_**b**_, which is a product of independent binomial distributions with parameters (*n*_*ij*_,*p*_*ij*_) for the treatment *j* in the *i*th study. For a given *Δ*, numerical algorithms can be used to find the maximum likelihood estimator of **p**_0_(*Δ*) to calculate $\widehat {G}(\Delta)$.

Similarly, the IS lower limit can be computed. It should be noted that designated statistics from the same model are used for the IS upper limit and the IS lower limit. For example, the asymptotic upper limit from the fixed-effects model is used as the designated statistic for the accurate upper limit, and then the lower limits from the same model is used for the accurate lower limit. We refer this accurate interval as the IS-F interval. When the asymptotic limits from the random-effects model are used as the designated statistics, the computed accurate limits are referred to be as the IS-R interval.

## Results

We first use an example from 18 schizophrenia clinical trials to illustrate the application of the proposed accurate intervals. In addition to the F interval, the R interval, the IS-F interval, and the IS-R interval, We also include the confidence interval for *Δ* by Tian [9] in the comparison (referred to be as the Tian interval). Tian interval can be computed by using their developed R function *meta*.*exact* from the *exactmeta* function, without the mid-p value approach. All data including studies with zero events are used in the confidence interval calculation.

These 18 schizophrenia clinical trials reported the number of all-cause mortality for patients treated with the long-acting injectable antipsychotics (LAI-AP) or the oral antipsychotics (OAP) which is the control treatment here. Data of these 18 trials are presented in Table 2, which was provided by Efthimiou [25]. Out of a total of 3774 participants treated with the LAI-AP, 7 events were observed. In the OAP group, there were 6 events recorded from a total of 2145 participants in the control group. The naive estimates for all-cause mortality rates are 0.185% and 0.279% in the LAI-AP group and the OAP group, respectively.

**Table 2:** Data from 18 clinical trials comparing all-cause mortality rate of patients treated with long-acting injectable antipsychotics (LAI-AP) or the oral antipsychotics (OAP) treatment as the control

	LAI-AP group		OAP group		Sample sizes	
Study	Events	Non-events	Events	Non-events	*N*_1_	*N*_2_
Kane 2012	1	268	0	134	269	134
Kane 2014	0	168	0	172	168	172
Meltzer 2015	0	415	1	206	415	207
Hirsch 1973	0	41	0	40	41	40
Jolley 1990	2	25	0	27	27	27
Odejide 1952	0	35	1	34	35	35
Rifkin 1977	0	23	1	21	23	22
Lauriello 2008	0	306	0	98	306	98
Berwaerts 2015	0	160	0	145	160	145
Fu 2015	2	162	0	170	164	170
Gopal 2010	0	221	0	135	221	135
Hough 2010	0	206	0	204	206	204
Kramer 2010	0	163	0	84	163	84
Nasrallah 2010	1	390	1	126	391	127
Pandinda 2010	1	487	0	164	488	164
Takahasji 2013	0	160	1	163	160	164
Kane 2003	0	302	1	97	302	98
Nasser 2016	0	235	0	119	235	119

Table 3 presents the estimated $\widehat {\Delta }$ and the 95% confidence interval for *Δ* using the five methods. The point estimate of $\widehat {\Delta }$ from the R method is similar to the Tian method, and they are larger than that from the F method. It can be seen that the Tian interval is much wider than others, and the asymptotic F or R intervals have shorter lengths than the proposed accurate intervals. The upper limits of the proposed accurate intervals are smaller than those of other intervals. All the intervals contain zero. Therefore, we fail to reject the null hypothesis that there is no difference between the LAI-AP treatment and the OAP treatment with regards to the all-cause mortality rate.

**Table 3:** Confidence intervals for risk difference between the LAI-AP group and the OAP group

		Asymptotic intervals			IS intervals			Tian interval		
Method	$\widehat \Delta $	lower	upper	length	lower	upper	length	lower	upper	length
Fixed-effects	-0.064%	-0.346%	0.218%	0.564%	-0.506%	0.165%	0.704%			
Random-effects	-0.030%	-0.382%	0.322%	0.671%	-0.509%	0.250%	0.758%			
Tian method	-0.028%							-0.843%	0.430%	1.273%

### Simulation studies

We conduct extensive simulation studies to compare coverage probability and average length of the five intervals: the F interval, the R interval, the IS-F interval, the IS-R interval, and the Tian interval. The nominal confidence level is set as 95%. The sample sizes, *n*_*ij*_, are assumed to be the same as those in the aforementioned example, as *N*_1_ and *N*_0_ in Table 2. The number of responses *X*_*ij*1_ follows a binomial distribution (*n*_*ij*_,*p*_*ij*_). We simulate *D*=1,000 data for each configuration: **Y**_1_,**Y**_2_,⋯, and **Y**_*D*_. For the proposed IS intervals, we generate *B*=2,000 importance samples from the estimated distribution using each simulated data.

Coverage probability is defined as the proportion of the pre-specified risk difference *Δ* being included in the confidence intervals:

$$CP=\frac{1}{D}\sum_{d=1}^{D} I\Big(\Delta \in CI(\mathbf{Y}_{\mathbf{d}})\Big).$$

A confidence interval with the simulated interval being closer to the nominal level is preferable. Average length is defined as the average of all the lengths

$$AL=\sum_{d=1}^{D} \frac{CI_{upper}(\mathbf{Y}_{\mathbf{d}})-CI_{lower}(\mathbf{Y}_{\mathbf{d}})}{D},$$

where *CI*_*lower*_ and *CI*_*upper*_ are the lower limit and the upper limit of an interval. When two intervals are comparable with regards to coverage probability, the one with a shorter average length outperforms the other.

#### Homogeneity of study effects

**Fig. 1:**
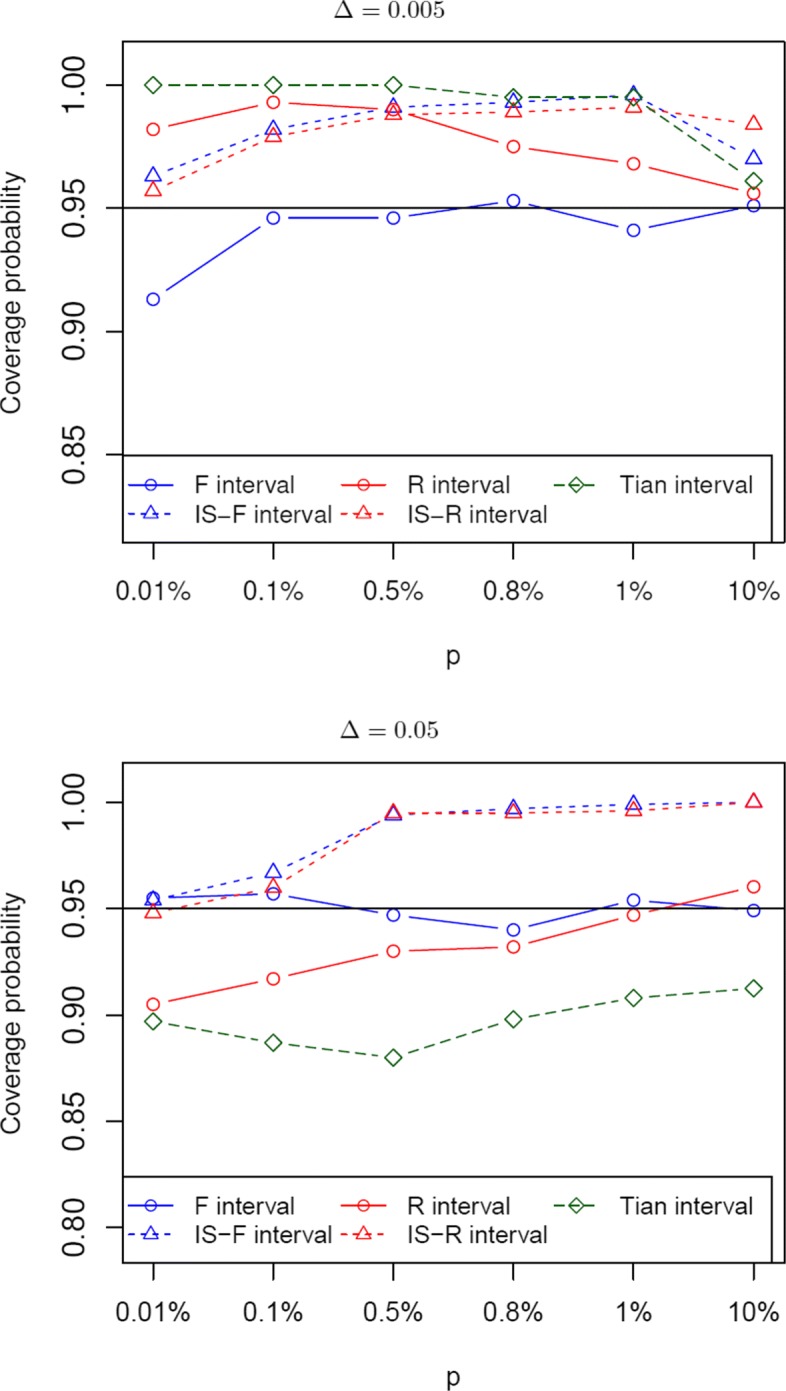
Coverage probability of the five methods under the study homogeneity assumption, with fixed and common rate *p*_*i*0_=*p* in the control group

We first compare the coverage probabilities of the five methods with fixed probabilities, **p**_1_ and **p**_0_. For simplicity, we assume a common rate in the control group, *p*_*i*0_=*p*, with *p* from 0.01% to 10%. The treatment probability is *p*_*i*1_=*p*+*Δ*. For each configuration of (*p*,*Δ*), the coverage probabilities of these methods are computed, see Fig. 1 when *Δ*=0.005 and 0.05. It can be seen that the F method has the coverage closer to the nominal level when *Δ*=0.005, except the case in which *p* is very low. As *Δ* is increased to 0.05, the F interval, the IS-R interval, and the IS-F interval have similar coverages when *p* is small. The IS-F interval and the IS-R interval are conservative when *p* is large. In this plot with *Δ*=0.05, the Tian interval and the R interval have the coverage probabilities below the nominal level. Overall, the F interval has good performance with regards to coverage when studies are homogeneous and have common rates.

Given the number of nuisance probabilities, it is difficult to compare the performance of the five methods under each configuration. With 18 studies and 5 considered probabilities, the number of possible configurations is 5^36^, which is over 10^25^. For this reason, we follow the approach by Tian et al. [9] to compare the performance of these methods by simulating the probabilities of the control group (**p**_0_) from uniform distributions: *U*(0,*b*), where *b*=0.0001, 0.001, 0.01, and 0.1. We consider the following five *Δ* values: 0.001, 0.005, 0.01, 0.05, and 0.1. Under the study homogeneity assumption, the probabilities of the treatment group **p**_1_ are then obtained as *p*_*i*1_=*p*_*i*0_+*Δ*.

Table 4 presents coverage and average length comparison between the five intervals when **p**_0_∼*U*(0,0.01*%*). Coverage probabilities of the F interval range from 89% to 96%. The R interval is very conservative when *Δ* is small, and its coverage is below 95% when *Δ* is larger. The Tian interval is conservative when *Δ*≤1*%*, but it could be as low as 76% when *Δ* is 10%. The proposed accurate intervals always have the coverage probabilities close to the nominal level as compared to the existing intervals. Average length is always an increasing function of *Δ* for each confidence interval method. The Tian intervals are wider than others when they all guarantee the coverage probability. The IS-R interval generally has a shorter length as compared to the R interval and the IS-F intervals.

**Table 4:** Coverage probability and average length comparison between the five intervals when **p**_0_∼ U(0,0.01%)

	The F interval		The IS-F interval		The R interval		The IS-R interval		The Tian interval	
*Δ*	Coverage	Length	Coverage	Length	Coverage	Length	Coverage	Length	Coverage	Length
0.1%	0.890	0.21%	0.968	0.36%	1.000	0.63%	0.984	0.34%	0.987	1.02%
0.5%	0.932	0.47%	0.970	0.71%	0.987	0.72%	0.957	0.67%	0.997	1.10%
1.0%	0.949	0.66%	0.958	0.83%	0.877	0.84%	0.944	0.76%	0.994	1.24%
5.0%	0.949	1.45%	0.946	1.45%	0.905	1.58%	0.952	1.46%	0.903	1.52%
10.0%	0.962	1.99%	0.962	2.00%	0.935	2.15%	0.960	2.00%	0.730	1.63%

**Fig. 2:**
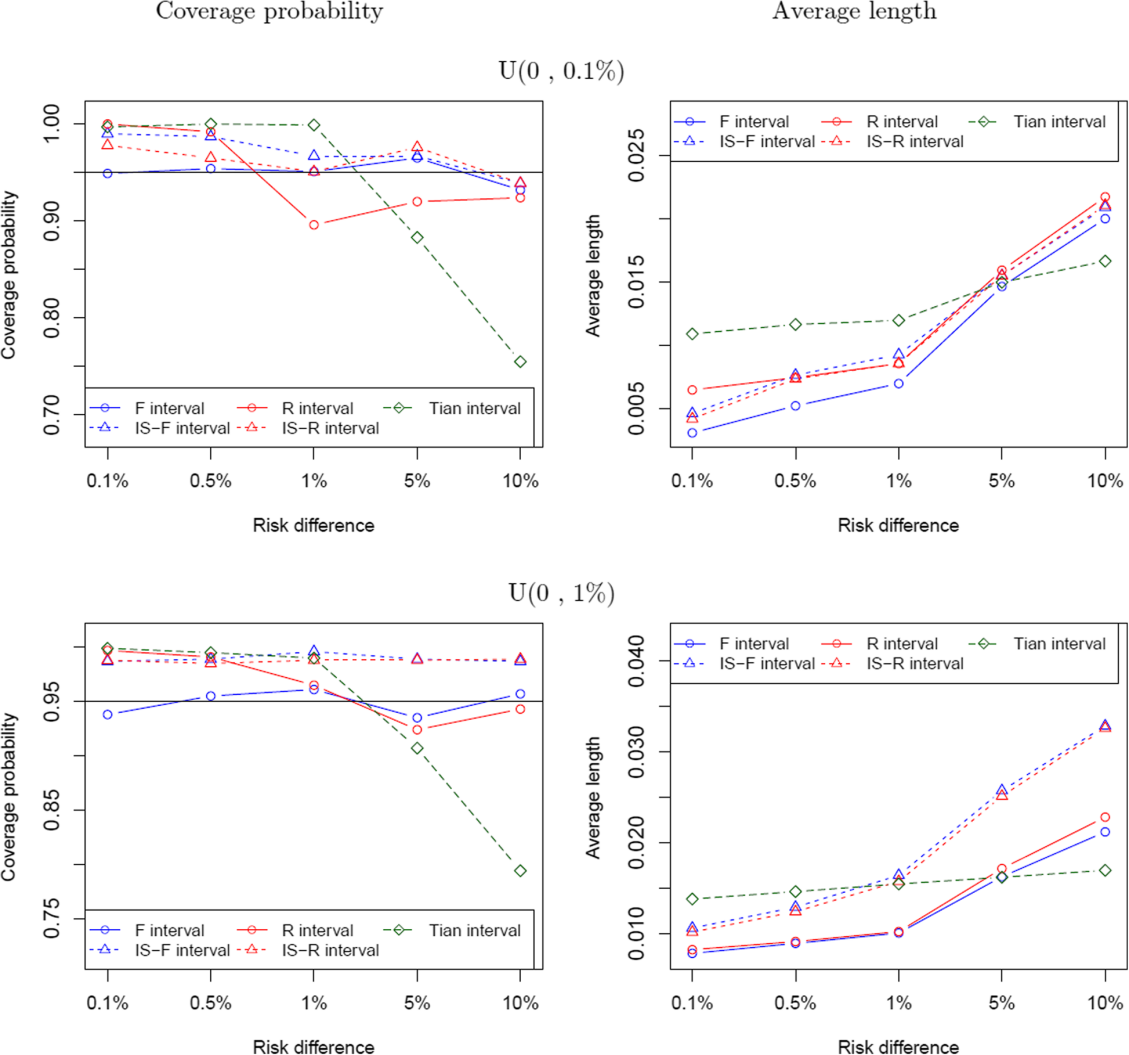
Coverage probability and average length comparison between the five intervals under the study homogeneity assumption, when the probability of the control group **p**_0_∼ U(0, 0.1%) and U(0, 1%)

When the event rates of the control group are higher with **p**_0_∼*U*(0,0.1*%*) in Fig. 2, the F interval generally performs better than others with regards to coverage probability and average length. When *Δ* is large (e.g., 10%), coverage probabilities of these intervals are all slightly below 95%. In this case with a small **p**_0_ and a relatively large *Δ*, the proposed intervals (IS-R or IS-F intervals) have better coverage probabilities than the F interval, and the length difference between the accurate intervals and the F interval is small. When *Δ*=10*%*, the coverage probability of the Tian interval is below 80%. When the rates are even higher with **p**_0_∼*U*(0,1*%*), the rates are not rare in these configurations, and the F interval outperforms others as seen in Fig. 2.

#### Heterogeneity of study effects

**Fig. 3:**
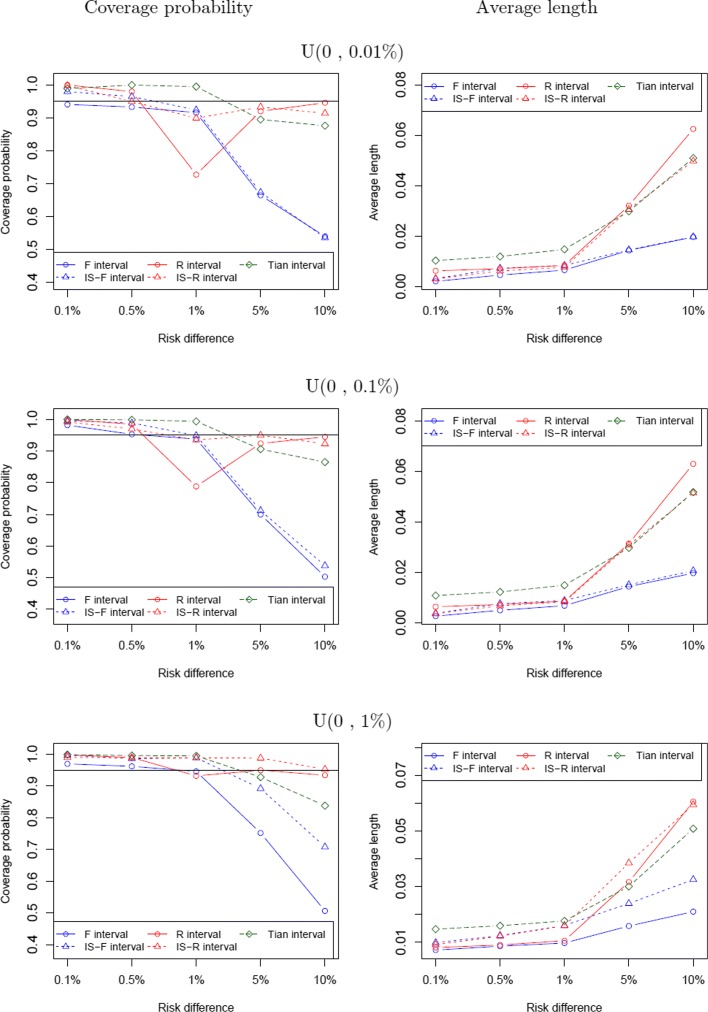
Coverage probability and average length comparison between the five intervals under the study heterogeneity assumption, when **p**_0_∼ U(0, 0.01%), U(0, 0.1%) and U(0, 1%)

Under the study heterogeneity assumption, the probability in the treatment group is *p*_*i*1_= *p*_*i*0_+*u*_*i*_, where *u*_*i*_ is the random study effect that follows a normal distribution with mean of *Δ* and standard deviation of *Δ*/2. Figure 3 presents the coverage probability and average length comparison between the five intervals when **p**_0_∼*U*(0,0.01*%*),*U*(0,0.1*%*), and *U*(0,1*%*). As *Δ* increases, the standard deviation of the probabilities in the treatment group goes up. When *Δ* is small, the F interval, the IS-R interval, and the IS-F interval have the coverage probabilities closer to the nominal level as compared to the R interval and the Tian interval. Coverage probabilities of the F interval and the IS-F interval drop to almost 50% when *Δ* is 10%. The R interval generally has good coverage when *Δ* is large. However, the R interval’s coverage probabilities are very low when *Δ*=1*%* in meta-analysis with rare events (e.g., **p**_0_∼*U*(0,0.01*%*) or *U*(0,0.1*%*)). The IS-R interval has consistent good performance with regards to coverage and length as compared to others in meta-analysis with rare events. Figure 3 also presents the results when the event rates are not rare (e.g., **p**_0_∼*U*(0,1*%*)). When *Δ* is large, the R interval and the IS-R interval have better coverage probabilities than others. When variance of study effects is small (for the configurations with small *Δ* values), the F interval performs better where the configurations are similar to the ones under the study homogeneity assumption.

## Conclusions

We propose using importance sampling to construct confidence intervals for risk difference in meta-analysis with rare events. The traditionally used F interval has satisfactory performance with regards to coverage probability and interval length when the rate of events is not rare under the study homogeneity assumption, but this interval could have a very low coverage probability under the study heterogeneity assumption. The IS-R interval based on the asymptotic limits from the random-effects model outperforms the existing intervals under the heterogeneity assumption. The IS intervals use the existing asymptotic limits to order the sample space. Although the asymptotic limits are computed from asymptotic approaches whose performances are based on the approximation of the test statistic to the limiting distribution, the order of these limits provides a useful information to produce better IS limits.

The Tian interval often guarantees the coverage probability when the rates of both groups are rare, but that interval could have the coverage probability below the nominal level when *Δ* is large. Theoretically, the Tian interval can be used as a designated statistic to order the sample space. However, simulations are involved in the Tian interval calculation that would significantly increase the computational intensity of the proposed IS intervals. In addition, the ordering of the sample space based on the Tian interval may change as the number of simulations being utilized. For these reasons, we do not include the IS intervals based on the ordering by the Tian interval.

## Discussion

The method by Buehler [13] to construct exact one-sided confidence interval is ideal for binary outcome when the size of the sample size is not too large that allows a full enumeration of the sample space [16*,*^26^*–*29]. However, it is not feasible in meta-analysis as it is extremely difficult to save the sample space under the unconditional framework with sample size in each treatment group fixed. If the upper bound of the possible number of events can be determined and the size of the sample size is not too large, exact Buehler interval may be computed. Otherwise, an efficient search algorithm should be developed to order the sample space efficiently.

Exact confidence intervals are preferable for statistical inference. However, it is often computationally intensive, such as the aforementioned the exact interval by Buehler [28*,*^30^*–*32]. For these reasons, simulation based intervals are proposed for use in practice, including the proposed interval here, the Tian interval, and the interval based on confidence distribution [24*,*^33^*–*35]. It is still a big challenge in exact meta-analysis by enumerating all possible data, which becomes a big data problem with the requirement of huge memory and computational power.

In addition to risk difference, odd ratio and risk ratio are also used to measure the treatment effect. For studies with zero events in one or both treatment groups, the estimated risk difference is zero. However, the estimated ratios could be infinity [17*,*^36^*–*39]. In order to avoid this issue, an arbitrary small number (e.g., *ε*=0.5, 1) is often added to each cell in the data. The performance of the test statistics is affected by the chosen small value [6*,*^40^*–*42]. The added value *ε* also raises the question of whether the number of participants in a study should be *n*_*ij*_ or *n*_*ij*_+2*ε*. We consider this as future work to study the IS intervals for ratios.

## Data Availability

Not applicable. This is a manuscript to develop novel statistical approaches, therefore, no real data is involved.

## Abbreviations

GLMMS: Generalized linear mixed models
IS: Importance sampling
LAI-AP: Long-acting injectable antipsychotics
OAP: Oral antipsychotics
